# Supplementary material for: Effects of GHRH Deficiency and GHRH Antagonism on Emotional Disorders in Mice
Source: Cells. 2023 Nov 12;12(22):2615. doi: 10.3390/cells12222615 (PMC10670114; doi:10.3390/cells12222615)
Supplement: Supplementary file 1 [file cells-12-02615-s001.zip › cells-2665071-supplementary.pdf]

cells MDPI

Article

Effects of GHRH Deficiency and GHRH Antagonism on Emotional Disorders in Mice

Lucia Recinella [1,†], Maria Loreta Libero [1,2,*], Serena Veschi [1,†], Anna Piro [1], Guya Diletta Marconi [3], Francesca Diomede [3], Annalisa Chiavaroli [1], Giustino Orlando [1], Claudio Ferrante [1], Rosalba Florio [1], Alessia Lamolinara [4], Renzhi Cai [5,6,7], Wei Sha [5,6,7], Andrew V. Schally [5,6,7], Roberto Salvatori [8], Luigi Brunetti [1,*] and Sheila Leone [1]

1 Department of Pharmacy, "G. d'Annunzio" University of Chieti-Pescara, 66013 Chieti, Italy; lucia.recinella@unich.it (L.R.); veschi@unich.it (S.V.); anna.piro@unich.it (A.P.); annalisa.chiavaroli@unich.it (A.C.); giustino.orlando@unich.it (G.O.); claudio.ferrante@unich.it (C.F.); rosalba.florio@unich.it (R.F.); sheila.leone@unich.it (S.L.)
2 Department of Cell Biology, Physiology and Immunology, University of Cordoba, 14014 Cordoba, Spain
3 Department of Innovative Technologies in Medicine & Dentistry, "G. d'Annunzio" University of Chieti-Pescara, 66013 Chieti, Italy; guya.marconi@unich.it (G.D.M.); francesca.diomede@unich.it (F.D.)
4 Department of Neuroscience Imaging and Clinical Sciences, "G. d'Annunzio" University of Chieti-Pescara, 66013 Chieti, Italy; alessia.lamolinara@unich.it
5 Veterans Affairs Medical Center, Miami, 33125 FL, USA; renzi.c@hotmail.com (R.C.); weisha17@gmail.com (W.S.); aschally@med.miami.edu (A.V.S.)
6 Division of Endocrinology, Diabetes and Metabolism, Division of Medical/Oncology, Department of Medicine, Department of Pathology, Miller School of Medicine, University of Miami, Miami, 33136 FL, USA
7 Sylvester Comprehensive Cancer Center, Miami, 33136 FL, USA
8 Department of Medicine, Division of Endocrinology, Diabetes and Metabolism, Johns Hopkins University School of Medicine, Baltimore, 21205 MD, USA; salvator@jhmi.edu
* Correspondence: maria.libero@unich.it (M.L.L.); luigi.brunetti@unich.it (L.B.)
† These authors contributed equally to this work.

Citation: Recinella, L.; Libero, M.L.; Veschi, S.; Piro, A.; Marconi, G.D.; Diomede, F.; Chiavaroli, A.; Orlando, G.; Ferrante, C.; Florio, R.; et al. Effects of GHRH Deficiency and GHRH Antagonism on Emotional Disorders in Mice. *Cells* **2023**, *12*, 2615. https://doi.org/10.3390/cells12222615

Academic Editor: Markus Fendt

Received: 29 September 2023
Revised: 6 November 2023
Accepted: 7 November 2023
Published: 12 November 2023

Copyright: © 2023 by the authors. Licensee MDPI, Basel, Switzerland. This article is an open access article distributed under the terms and conditions of the Creative Commons Attribution (CC BY) license (https://creativecommons.org/licenses/by/4.0/).

Abstract: Growth hormone (GH)-releasing hormone (GHRH) has been suggested to play a crucial role in brain functions. We aimed to further investigate the effects of a novel GHRH antagonist of the Miami (MIA) series, MIA-602, on emotional disorders and explore the relationships between endocrine system and mood disorders. In this context, the effects induced by MIA-602 were also analyzed in comparison to vehicle-treated mice with GH deficiency due to generalized ablation of the GHRH gene [GHRH knock out (GHRHKO)]. We show that chronic subcutaneous administration of MIA-602 to wild type (+/+) mice, as well as generalized ablation of the GHRH gene, are associated with anxiolytic and antidepressant behavior. Moreover, immunohistochemical and western blot analyses suggested an evident activation of Nrf2, HO1, and NQO1 in prefrontal cortex of both +/+ mice treated with MIA-602 (+/+ MIA-602) and homozygous GHRHKO (-/- control) animals. Finally, we also found significantly decreased COX-2, iNOS, NFkB, and TNF-α gene expressions, as well as increased P-AKT and AKT levels in +/+ MIA-602 and -/- control animals compared to +/+ mice treated with vehicle (+/+ control). We hypothesize the generalized ablation of the GHRH gene leads to a dysregulation of neural pathways, which is mimicked by GHRH antagonist treatment.

Keywords: GHRH deficiency; GHRH antagonism; anxiety; depression; mood disorders

Materials and Methods

Haematoxylin-eosin staining/light microscopy analysis and immunohistochemistry

Mouse prefrontal cortex and hippocampus were fixed in 10% phosphate-buffered formalin for 2.5 hours. Each tissue block was dehydrated in a series of alcohol solutions of 50%, 70%, 96% and 99% and then in Bioclear. Samples were then paraffin-embedded and cut into 7 µm-thick sections. Sections were de-waxed (Bioclear and alcohol in progressively lower concentrations), rehydrated and processed for haematoxylin-eosin and for anti-Nrf2 immunohistochemical analysis according to manufacturer's protocol [1]. In the carotid body, galanin is a signal for neurogenesis in young, and for neurodegeneration in the old and in drug-addicted subjects). Primary antibody anti-Nfr2 (rabbit polyclonal, sc-722, Santa Cruz Biotechnology, CA, USA) was applied for 2 hours at room temperature and diluted 1:200 in PBS (1x). The immunohistochemical reactions were revealed with Rabbit specific HRP/DAB detection IHC kit (ab236469). Peroxidase reaction was developed using diaminobenzidine (DAB) chromogen and nuclei were counterstained with haematoxylin. Lastly, sections were dehydrated, cleared with Bioclear and mounted in Eukitt mounting medium (Bio Optica, Milano, Italy). Negative control was performed by omitting the primary antibody. Samples were then observed by means of LEICA DM 4000 B light microscopy (Leica Cambridge Ltd., Cambridge, UK) equipped with a Leica DFC 320 camera (Leica Cambridge Ltd.) for computerized images.

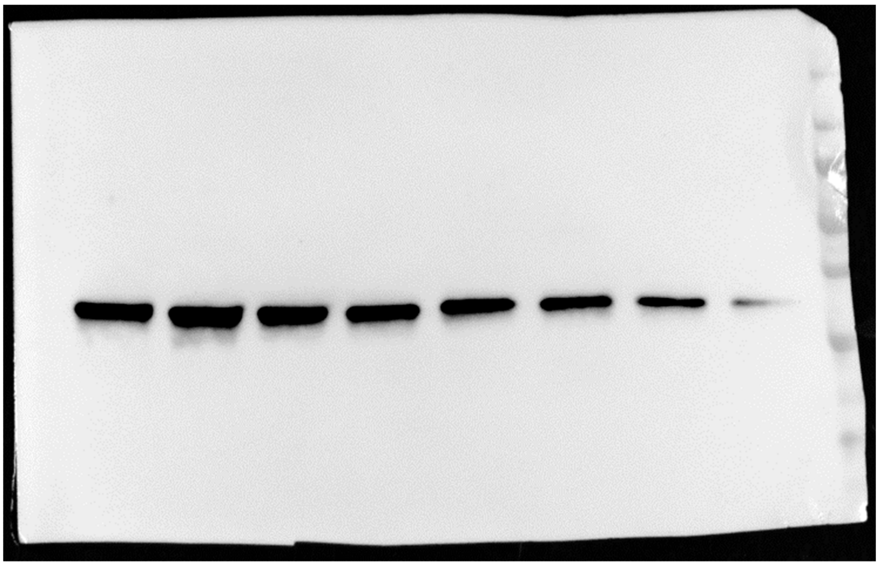

Supplementary Figure S1. Haematoxylin-eosin staining and immunohistochemical analysis of Nrf2 in mice prefrontal cortex. Morphological features and detection of nuclear factor erythroid 2-related factor 2 (Nrf2) in prefrontal cortex have been analyzed by hematoxylin-eosin (H&E) staining and immunohistochemistry, respectively. H&E stained sections of (a) +/+ MIA-602, (b) -/- vehicle-treated (-/- control), and (c) +/+ vehicle-treated (+/+ control) showed the normal histological structure of the prefrontal cortex. Immunohistochemical examination revealed positive immunostaining for Nrf2 expression in (d) +/+ MIA-602 and (e) -/- control + compared to (f) +/+ control +.

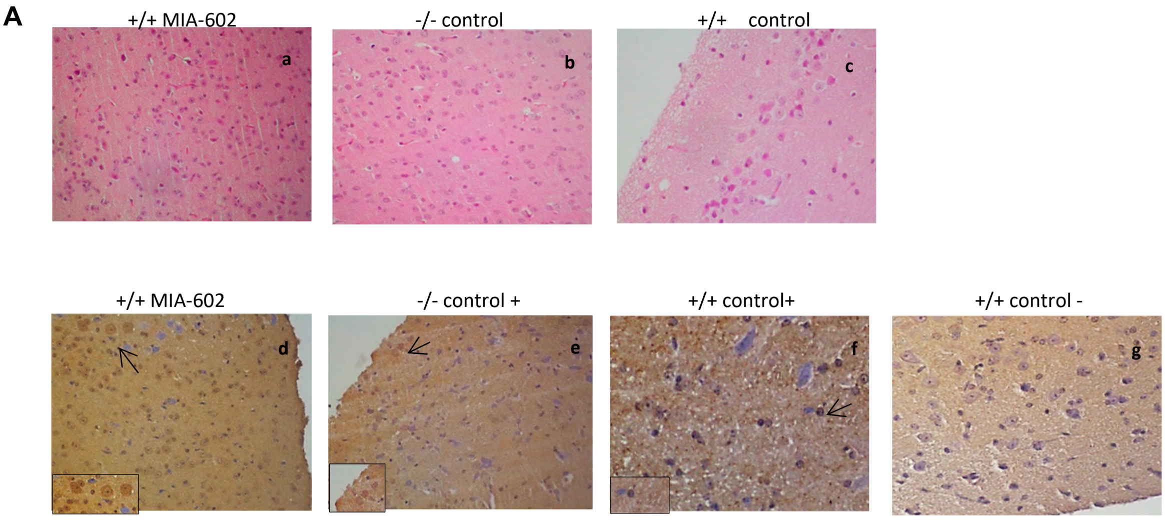

Western blot analysis

Cortex tissue was collected from mice treated or not with MIA-602. Cerebral samples were homogenized by Ultra-Turrax homogenizer (IKA-Werke, Staufen, Germany) in RIPA buffer supplemented with 1mM PMSF (Phenylmethanesulfonyl Fluoride) and protease and phosphatase inhibitors cocktails (Sigma, St. Louis, MO, USA). Lysed samples were sonicated and centrifuged at 15,000 rpm (4 °C for 20 min). Protein concentrations were quantified by the BCA Protein Assay (Thermo Scientific, Rockford, IL, USA) and 40 µg of protein lysates were subjected to electrophoresis followed by immunoblotting [2]. The nitrocellulose membranes were blocked in 5% nonfat dry milk and incubated overnight at 4 °C with the appropriate primary antibodies. Anti-TrkB and anti-HO-1 rabbit monoclonal antibodies were purchased from Cell Signaling Technology, Inc. (Beverly, MA, USA). Goat polyclonal anti-NQO1, anti-P-AKT, anti-AKT, anti-PI3K antibody were obtained from Abcam (Cambridge, England). The membranes were then incubated with either anti-rabbit or anti-mouse HRP-conjugated secondary antibodies (Cell Signaling Technology, Beverly, MA, USA). The blots were revealed with the Westar ηC Ultra 2.0 chemiluminescence substrate (Cyanagen, Bologna, Italy). GAPDH was used as loading control (Santa Cruz Biotechnology, Dallas, TX, USA).

Supplementary Figure S2. Effects of MIA-602 on expression of heme oxygenase-1 (HO1) in prefrontal cortex. Mice were treated daily for 4 weeks by subcutaneous administration of the GHRH antagonist MIA-602 (5 µg) or vehicle solution. Cortex was collected from mice treated or not with MIA-602. Cerebral samples were homogenized by Ultra-Turrax homogenizer (IKA-Werke, Staufen, Germany) in RIPA buffer supplemented with 1mM PMSF (Phenylmethanesulfonyl Fluoride) and protease and

phosphatase inhibitors cocktails (Sigma, St. Louis, MO, USA). Lysed samples were sonicated and centrifuged at 15,000 rpm (4 °C for 20 min). Protein concentrations were quantified by the BCA Protein Assay (Thermo Scientific, Rockford, IL, USA) and 40 µg of protein lysates were subjected to electrophoresis followed by immunoblotting. The nitrocellulose membranes were blocked in 5% nonfat dry milk and incubated overnight at 4 °C with the appropriate primary antibodies. Anti-HO-1 rabbit monoclonal antibodies was purchased from Cell Signaling Technology, Inc. (Beverly, MA, USA). The membranes were then incubated with either anti-rabbit or anti-mouse HRP-conjugated secondary antibodies (Cell Signaling Technology, Beverly, MA, USA). The blots were revealed with the Westar ηC Ultra 2.0 chemiluminescence substrate (Cyanagen, Bologna, Italy). GAPDH was used as loading control (Santa Cruz Biotechnology, Dallas, TX, USA).

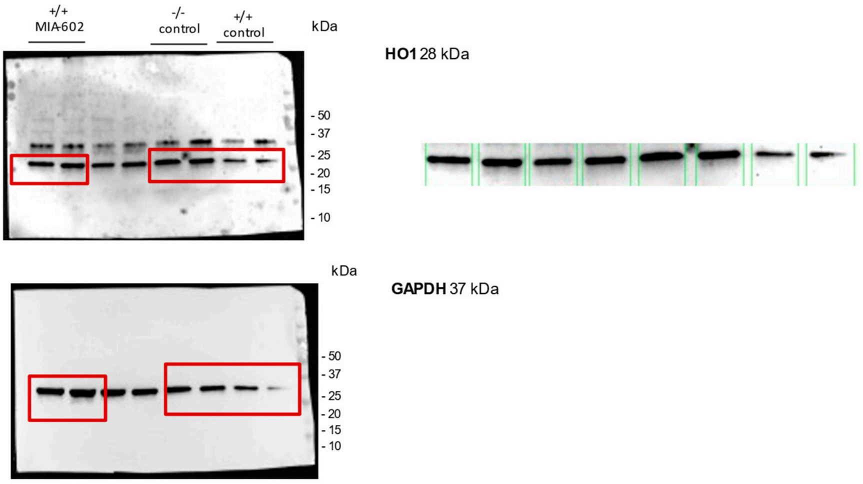

Supplementary Figure S3. Effects of MIA-602 on expression of NAD(P)H quinone oxidoreductase 1 (NQO1) in prefrontal cortex. Mice were treated daily for 4 weeks by subcutaneous administration of the GHRH antagonist MIA-602 (5 µg) or vehicle solution. Cortex and hippocampus tissues were collected from mice treated or not with MIA-602. Prefrontal cortex was homogenized by Ultra-Turrax homogenizer (IKA-Werke, Staufen, Germany) in RIPA buffer supplemented with 1mM PMSF (Phenylmethanesulfonyl Fluoride) and protease and phosphatase inhibitors cocktails (Sigma, St. Louis, MO, USA). Lysed samples were sonicated and centrifuged at 15,000 rpm (4 °C for 20 min). Protein concentrations were quantified by the BCA Protein Assay (Thermo Scientific, Rockford, IL, USA) and 40 µg of protein lysates were subjected to electrophoresis followed by immunoblotting. The nitrocellulose membranes were blocked in 5% nonfat dry milk and incubated overnight at 4 °C with the appropriate primary antibodies. Goat polyclonal anti-NQO1 antibody was obtained from Abcam (Cambridge, England). The membranes were then incubated with either anti-rabbit or anti-mouse HRP-conjugated secondary antibodies (Cell Signaling Technology, Beverly, MA, USA). The blots were revealed with the Westar ηC Ultra 2.0 chemiluminescence substrate (Cyanagen, Bologna, Italy). GAPDH was used as loading control (Santa Cruz Biotechnology, Dallas, TX, USA).

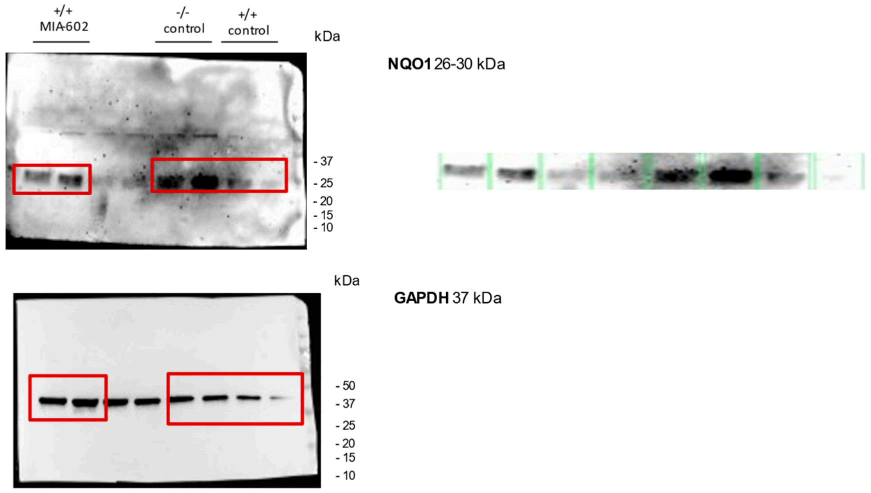

Supplementary Figure S4. Effects of MIA-602 on expression of TrkB in prefrontal cortex. Mice were treated daily for 4 weeks by subcutaneous administration of the GHRH antagonist MIA-602 (5 µg) or vehicle solution. Cortex and hippocampus tissues were collected. Prefrontal cortex was homogenized by Ultra-Turrax homogenizer (IKA-Werke, Staufen, Germany) in RIPA buffer supplemented with 1mM PMSF (Phenylmethanesulfonyl Fluoride) and protease and phosphatase inhibitors cocktails (Sigma, St. Louis, MO, USA). Lysed samples were sonicated and centrifuged at 15,000 rpm (4 °C for 20 min). Protein concentrations were quantified by the BCA Protein Assay (Thermo Scientific, Rockford, IL, USA) and 40 µg of protein lysates were subjected to electrophoresis followed by immunoblotting. The nitrocellulose membranes were blocked in 5% nonfat dry milk and incubated overnight at 4 °C with the appropriate primary antibodies. Anti-TrkB monoclonal antibody was purchased from Cell Signaling Technology, Inc. (Beverly, MA, USA). The membranes were then incubated with either anti-rabbit or anti-mouse HRP-conjugated secondary antibodies (Cell Signaling Technology, Beverly, MA, USA). The blots were revealed with the Westar ηC Ultra 2.0 chemiluminescence substrate (Cyanagen, Bologna, Italy). GAPDH was used as loading control (Santa Cruz Biotechnology, Dallas, TX, USA).

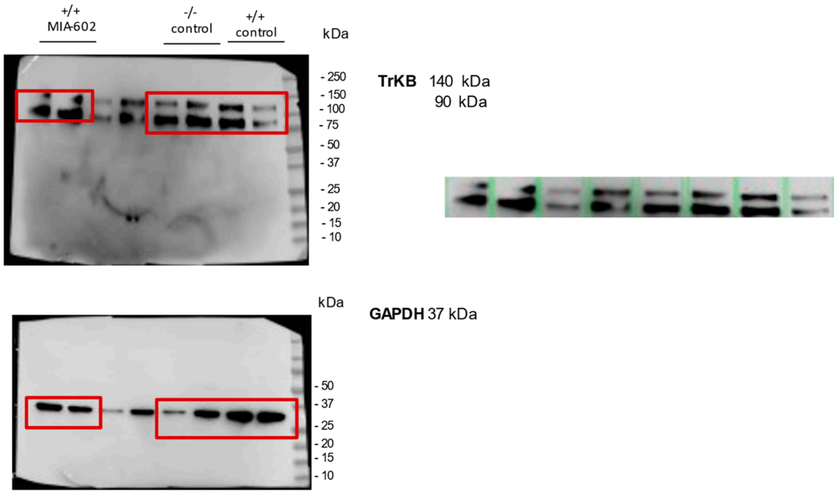

Supplementary Figure S5. Effects of MIA-602 on expression of P-AKT in prefrontal cortex. Mice were treated daily for 4 weeks by subcutaneous administration of the GHRH antagonist MIA-602 (5 µg) or vehicle solution. Prefrontal cortex was homogenized by Ultra-Turrax homogenizer (IKA-Werke, Staufen, Germany) in RIPA buffer supplemented with 1mM PMSF (Phenylmethanesulfonyl Fluoride) and protease and phosphatase inhibitors cocktails (Sigma, St. Louis, MO, USA). Lysed samples were sonicated and centrifuged at 15,000 rpm (4 °C for 20 min). Protein concentrations were quantified by the BCA Protein Assay (Thermo Scientific, Rockford, IL, USA) and 40 µg of protein lysates were subjected to electrophoresis followed by immunoblotting. The nitrocellulose membranes were blocked in 5% nonfat dry milk and incubated overnight at 4 °C with the appropriate primary antibodies. Anti-P-AKT monoclonal antibody was purchased from Cell Signaling Technology, Inc. (Beverly, MA, USA). The membranes were then incubated with either anti-rabbit or anti-mouse HRP-conjugated secondary antibodies (Cell Signaling Technology, Beverly, MA, USA). The blots were revealed with the Westar ηC Ultra 2.0 chemiluminescence substrate (Cyanagen, Bologna, Italy). GAPDH was used as loading control (Santa Cruz Biotechnology, Dallas, TX, USA).

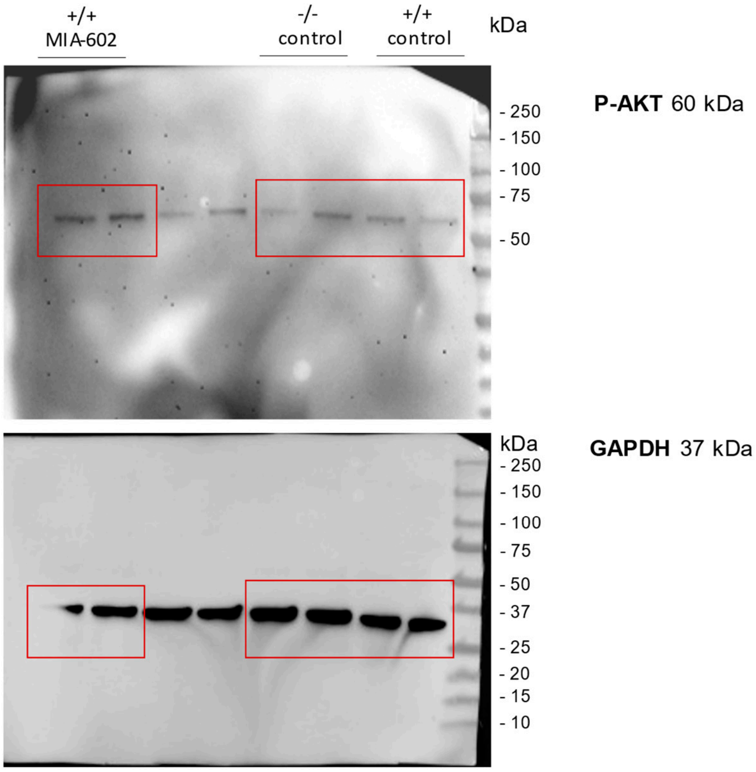

Supplementary Figure S6. Effects of MIA-602 on expression of AKT in prefrontal cortex. Mice were treated daily for 4 weeks by subcutaneous administration of the GHRH antagonist MIA-602 (5 µg) or vehicle solution. Prefrontal cortex was homogenized by Ultra-Turrax homogenizer (IKA-Werke, Staufen, Germany) in RIPA buffer supplemented with 1mM PMSF (Phenylmethanesulfonyl Fluoride) and protease and phosphatase inhibitors cocktails (Sigma, St. Louis, MO, USA). Lysed samples were sonicated and centrifuged at 15,000 rpm (4 °C for 20 min). Protein concentrations were quantified by the BCA Protein Assay (Thermo Scientific, Rockford, IL, USA) and 40 µg of protein lysates were subjected to electrophoresis followed by immunoblotting. The nitrocellulose membranes were blocked in 5% nonfat dry milk and incubated overnight at 4 °C with the appropriate primary antibodies. Anti-AKT monoclonal antibody was purchased from Cell Signaling Technology, Inc. (Beverly, MA, USA). The membranes were then incubated with either anti-rabbit or anti-mouse HRP-conjugated secondary antibodies

(Cell Signaling Technology, Beverly, MA, USA). The blots were revealed with the Westar ηC Ultra 2.0 chemiluminescence substrate (Cyanagen, Bologna, Italy). GAPDH was used as loading control (Santa Cruz Biotechnology, Dallas, TX, USA).

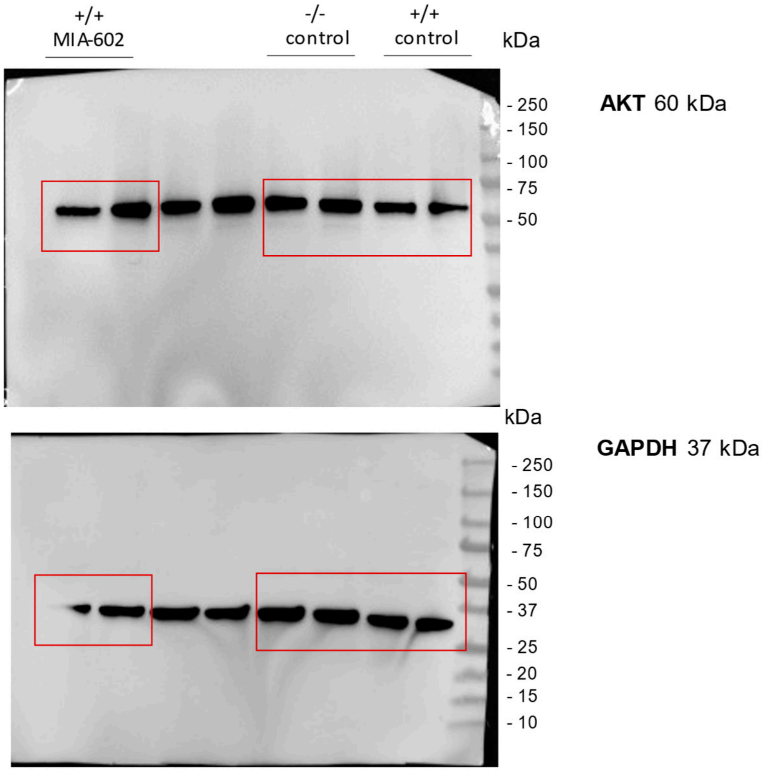

Supplementary Figure S7. Effects of MIA-602 on expression of PI3K in prefrontal cortex. Mice were treated daily for 4 weeks by subcutaneous administration of the GHRH antagonist MIA-602 (5 µg) or vehicle solution. Prefrontal cortex was homogenized by Ultra-Turrax homogenizer (IKA-Werke, Staufen, Germany) in RIPA buffer supplemented with 1mM PMSF (Phenylmethanesulfonyl Fluoride) and protease and phosphatase inhibitors cocktails (Sigma, St. Louis, MO, USA). Lysed samples were sonicated and centrifuged at 15,000 rpm (4 °C for 20 min). Protein concentrations were quantified by the BCA Protein Assay (Thermo Scientific, Rockford, IL, USA) and 40 µg of protein lysates were subjected to electrophoresis followed by immunoblotting. The nitrocellulose membranes were blocked in 5% nonfat dry milk and incubated overnight at 4 °C with the appropriate primary antibodies. Anti-PI3K monoclonal antibody was purchased from Cell Signaling Technology, Inc. (Beverly, MA, USA). The membranes were then incubated with either anti-rabbit or anti-mouse HRP-conjugated secondary antibodies (Cell Signaling Technology, Beverly, MA, USA). The blots were revealed with the Westar ηC Ultra 2.0 chemiluminescence substrate (Cyanagen, Bologna, Italy). GAPDH was used as loading control (Santa Cruz Biotechnology, Dallas, TX, USA).

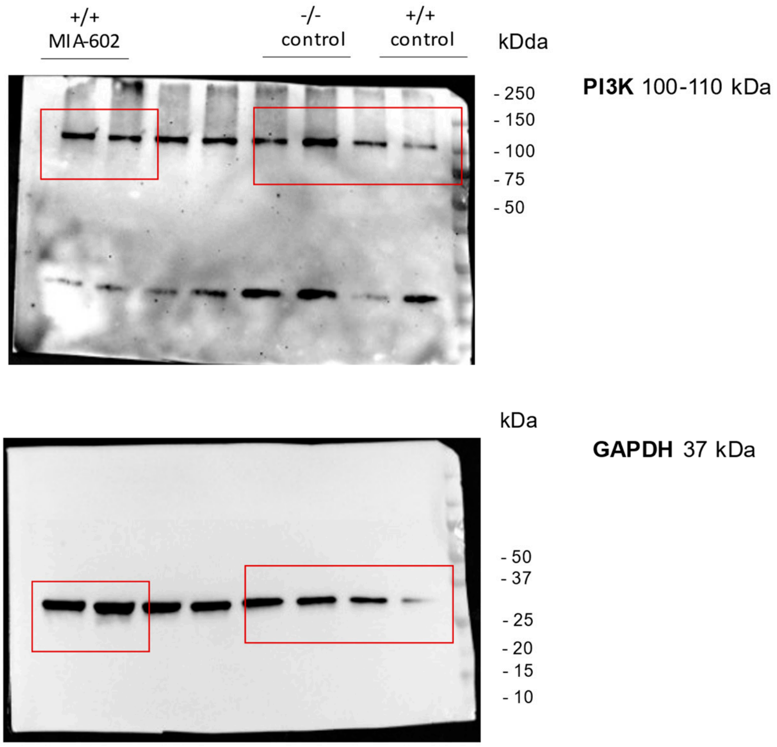

References

1. Di Giulio, C.; Marconi, G.D.; Zara, S.; Di Tano, A.; Porzionato, A.; Pokorski, M.; Cataldi, A.; Mazzatenta, A. Selective Expression of Galanin in Neuronal-Like Cells of the Human Carotid Body. *Adv Exp Med Biol.* **2015**, *860*, 315–323. doi: 10.1007/978-3-319-18440-1_36.
2. Veschi, S.; De Lellis, L.; Florio, R.; Lanuti, P.; Massucci, A.; Tinari, N.; De Tursi, M.; di Sebastiano, P.; Marchisio, M.; Natoli, C.; Cama, A. Effects of Repurposed Drug Candidates Nitroxoline and Nelfinavir as Single Agents or in Combination with Erlotinib in Pancreatic Cancer Cells. *J Exp Clin Cancer Res* **2018**, *37*, 236, doi:10.1186/s13046-018-0904-2.

Disclaimer/Publisher's Note: The statements, opinions and data contained in all publications are solely those of the individual author(s) and contributor(s) and not of MDPI and/or the editor(s). MDPI and/or the editor(s) disclaim responsibility for any injury to people or property resulting from any ideas, methods, instructions or products referred to in the content.
